# Investigation of the impact of nonsynonymous mutations on thyroid peroxidase dimer

**DOI:** 10.1371/journal.pone.0291386

**Published:** 2023-09-12

**Authors:** Mst. Noorjahan Begum, Rumana Mahtarin, Sinthyia Ahmed, Imrul Shahriar, Shekh Rezwan Hossain, Md. Waseque Mia, Syed Saleheen Qadri, Firdausi Qadri, Kaiissar Mannoor, Sharif Akhteruzzaman

**Affiliations:** 1 Department of Genetic Engineering & Biotechnology, University of Dhaka, Dhaka, Bangladesh; 2 Institute for Developing Science and Health Initiatives (ideSHi), ECB Chattar, Mirpur, Dhaka, Bangladesh; 3 Virology Laboratory, Infectious Diseases Division, International Centre for Diarrhoeal Disease Research, Bangladesh, Mohakhali, Dhaka, Bangladesh; 4 Department of Biochemistry and Molecular Biology, Shahjalal University of Science and Technology, Sylhet, Bangladesh; 5 Division of Computer Aided Drug Design, The Red-Green Research Centre, BICCB, Tejgaon, Dhaka, Bangladesh; 6 Mucosal Immunology and Vaccinology, Infectious Diseases Division, International Centre for Diarrhoeal Disease Research, Bangladesh, Mohakhali, Dhaka, Bangladesh; National Institute of Health, INDIA

## Abstract

Congenital hypothyroidism is one of the most common preventable endocrine disorders associated with thyroid dysgenesis or dyshormonogenesis. Thyroid peroxidase (*TPO*) gene defect is mainly responsible for dyshormonogenesis; a defect in the thyroid hormone biosynthesis pathway. In Bangladesh, there is limited data regarding the genetic etiology of Congenital Hypothyroidism (CH). The present study investigates the impact of the detected mutations (p.Ala373Ser, and p.Thr725Pro) on the TPO dimer protein. We have performed sequential molecular docking of H_2_O_2_ and I^-^ ligands with both monomers of TPO dimer to understand the iodination process in thyroid hormone biosynthesis. Understanding homodimer interactions at the atomic level is a critical challenge to elucidate their biological mechanisms of action. The docking results reveal that mutations in the dimer severely disrupt its catalytic interaction with essential ligands. Molecular dynamics simulation has been performed to validate the docking results, thus realizing the consequence of the mutation in the biological system’s mimic. The dynamics results expose that mutations destabilize the TPO dimer protein. Finally, principal component analysis exhibits structural and energy profile discrepancies in wild-type and mutant dimers. The findings of this study highlight that the mutations in TPO protein can critically affect the dimer structure and loss of enzymatic activity is persistent. Other factors also might influence the hormone synthesis pathway, which is under investigation.

## 1. Introduction

Congenital hypothyroidism (CH) is an endocrine disorder that prevalently affects newborns. The most detrimental effect imposes on cognitive and motor development. The frequency of CH is more than twice (1 in 1300) the global occurrence rate (1 in 3000–4000) in Bangladesh, which raises a significant concern [1, 2]. Early screening and detection of gene mutations are indispensable to prevent the severity of congenital hypothyroidism [3]. The defect in thyroid hormone (TH) synthesis or thyroid dyshormonogenesis causes 10% to 20% of CH cases. Thyroid dyshormonogenesis is an inborn defect in genes coding proteins essential for TH synthesis, secretion, or recycling [4].

The mutations in the thyroid peroxidase (*TPO*) gene can severely affect thyroid hormone synthesis due to total iodide organification defects (TIOD) or partial iodide organification defects (PIOD) [3]. TPO exists as a homodimer protein (consisting of 1866 amino acid residues) in the biological system to exert its effect on thyroid hormone biosynthesis. It is a glycosylated heme protein found in the apical membrane of the thyrocyte, where it accomplishes both iodination and coupling of iodotyrosyl residues in thyroglobulin. TPO facilitates the synthesis of enzymatically active thyroid hormones, triiodothyronine (T3) and thyroxine (T4) [2, 3]. The functional diversity of TPO increases through a few post-transcriptional modifications, which are glycosylation, heme integration, propeptide deletion, and dimerization. At least partial folded human TPO protein (only 15–20%) can reach the cell surface while the proteasome rapidly degrades the unfolded molecules. The precise folding of TPO and attachment of heme is crucial for TPO to depart from the endoplasmic reticulum (ER) and reach the cell surface through intracellular trafficking [5]. Moreover, TPO turns into an active form in the presence of hydrogen peroxide [6]. In the cell line, the supplementation of H_2_O_2_ significantly promotes the autocatalytic covalent attachment of heme [5]. The catalytic center (heme-binding region) in the TPO protein is encoded by exons 7–11 of *TPO* gene, which is crucial for the enzymatic activity. Previous studies identified exons 7–14 as mutational hot spots in the *TPO* gene [3, 7].

As TPO homodimer catalyzes the iodination process in the biosynthesis of thyroid hormones, therefore, understanding the interactions and how they are affected by mutations are essential to investigate the severity of congenital hypothyroidism. The possible modes of TPO dimer structure through a molecular modeling approach has been carried out which suggested the consideration of the protein’s oligomeric state [8]. Conversely, although homodimers are abundant in nature, only a few samples are available for symmetric homodimers’ computational models [9]. However, the protein complexes, predominantly homomers, are generally organized through allosteric behavior and represent significant associations to predict mutations’ damaging effects [10].

Our previous *in silico* based study on TPO has detected three nonsynonymous mutations where the substitution mutations, p.Ala373Ser, and p.Thr725Pro had a more damaging impact on the TPO monomer protein considering its interaction with the heme molecule [2]. The present study is based on the mutations (p.Ala373Ser, and p.Thr725Pro) being detected in the thyroid peroxidase (*TPO*) gene from a sequence-based technique in our previous study [2]. The study aims to investigate the adverse effects of nonsynonymous mutations on the structural integrity and the enzymatic activity of the TPO dimer using molecular docking, non-covalent interaction, molecular dynamics, and principal component analysis.

## 2. Materials and methods

### 2.1. Dimer modeling of TPO WT HD, TPO MT1 HD, and TPO MT2 HD 3D structures

From our previous study, the best models of monomers [2] from I-TASSER (sequence of TPO gene retrieved from the NCBI database: Accession number; NC_000002.12) [2] were employed to model homodimers of the TPO wild type and mutants (TPO MT1 HD, and TPO MT2 HD) using the SymmDock web server. The mutant dimer models TPO MT1 HD, and TPO MT2 HD were designed based on mutations p.Ala373Ser, and p.Thr725Pro respectively. TPO full-length dimer proteins were modeled considering the better result of heme docking with TPO full-length monomer structures [2]. I-TASSER predicted previous monomer structures were validated by Verify3D server, and RAMPAGE server and the outcomes were shown in Table S1 of S1 File [2]. Also, we had analysed the 3D view of TPO monomer from AlphaFold server and SWISS-MODEL homology-modelling server. However, the the structures from I-TASSER were quite better in conformation than the other server (Fig S1 in S1 File).

From the 3D coordinates of I-TASSER predicted TPO monomers [2], SymmDock predicted the entire dimer complexes.

Generally, SymmDock provides complexes with Cn symmetry by geometry-based docking [11]. The reliable 3D structure determination in most of homo-oligomeric transmembrane proteins (HoTPs) depends on the protocol including cyclic symmetry (Cn symmetry). Moreover, ∼97% of 118 X-ray crystallographically solved structures of homo-oligomeric transmembrane proteins are Cn symmetric which highlights that the prevalence of Cn symmetric HoTPs and the benefits of integrating geometry restraints in quaternary structure determination [12]. Hence, we utilized SymmDock predicted homodimers of TPO proteins for the analyses [13].

### 2.2. Visualization of molecular interactions of heme with TPO WT HD, TPO MT1 HD, and TPO MT2 HD

The non-covalent interactions of heme with TPO WT HD, TPO MT1 HD, and TPO MT2 HD were visualized by BIOVIA Discovery Studio version 4.5. The interactions of heme were analyzed for “L (left)-monomer” and “R (right)-monomer” of the structures to realize the catalytic activity in each dimer. The constructed data table with non-covalent interactions was summarized in the results section.

### 2.3. Quantum mechanical (QM) calculations for optimization of hydrogen peroxide (H_2_O_2_) and iodide (I^-^)

The initial geometries of H_2_O_2_ and I^−^ structures were obtained from the PubChem database. We optimized the ligands through density functional theory (DFT) engaging Becke’s (B) exchange functional combining Lee, Yang, and Parr’s (LYP) correlation functional in Gaussian 09 program package. The 6-31G basis set was used to optimize H_2_O_2,_ and the MIDIX basis set was applied for iodide optimization (Fig S2 in S1 File). After ligand structure optimization, the bond distances, bond angles, structural integrity, orientation of the atoms in ligands, proper bonding among atoms were analyzed to get more accurate results of molecular docking (Table S2 in S1 File).

### 2.4. Sequential molecular docking approach for TPO WT HD, TPO MT1 HD, and TPO MT2 HD with hydrogen peroxide (H_2_O_2_) and iodide (I^-^) ligands

The heme bound TPO WT HD, TPO MT1 HD, and TPO MT2 HD 3D structures were retrieved after stabilization by 5000 ps MD simulation. The stable conformer of each dimer was sequentially docked with H_2_O_2_ and I^−^ ligands by AutoDock Vina program. By applying a sequential approach, the effects of mutations on TPO wild-type and mutant homodimer proteins’ catalytic activity were analyzed. Firstly, H_2_O_2_ was docked with one monomer of the dimer, and the monomer was denoted as the “L (left)–monomer.” After that, docking of H_2_O_2_ was performed on the next monomer, marked as the “R (right)–monomer.” Then, similar sequential docking was repeated for I^−^ ligand. After docking, the H_2_O_2_ and I^−^ interactions were visualized and analyzed for each monomer’s specific amino acids by using the BIOVIA Discovery version 4.5. The same procedures had been performed for all the three predicted dimer structures of TPO WT HD, TPO MT1 HD, and TPO MT2 HD, and the effects of the mutations on TPO dimer were observed by comparing the results. Grid box value center and grid box size for H_2_O_2_ and I^−^ docking with dimer structures were optimized around the catalytic site residues (Asp238, His239, Arg396, Glu399, and His494 residues) that were present in the active site of TPO i.e., MPO-like domain of each monomer and presented in Tables S3-S6 of S1 File.

### 2.5. Molecular dynamics (MD) simulation

The molecular dynamics simulation of TPO dimer model structures and dimer-ligand complexes was performed using the YASARA dynamics program [14], and AMBER14 force field [15] was employed for the calculations. The simulation system was equilibrated for 250 ps. During the equilibration phase, the membrane was artificially stabilized. The entire setting was equilibrated with 0.9% NaCl and water solvent at 310K temperature. The simulation temperature was regulated by the Berendsen thermostat process. The long-range electrostatic interactions were maintained through the Particle Mesh Ewald algorithm. The whole simulation was performed by applying a periodic boundary condition. The snapshots were collected at every 100 ps for 5000 ps MD simulation during the 1.25 fs time step for each structure. Subsequently, different data from MD simulation, including root mean square deviation (RMSD), root mean square fluctuation (RMSF) values, were collected and analyzed according to our former protocol [16–18].

Further, CABS-flex 2.0 web server was used for fast simulations of flexibility of protein structures [19]. This server is suitable for simulations of larger and multimeric proteins. The system generates an analogous representation of protein flexibility compared to all-atom MD [20] and NMR ensembles [21]. In CABS-flex, CABS coarse-grained protein model is used for protein dynamics simulation [22]. The CABS model applies the Monte Carlo dynamics and asymmetric Metropolis scheme which achieves the necessities of microscopic reversibility and Boltzmann distribution of the resulted ensembles.

### 2.6. Principal component analysis (PCA)

Principal component analysis (PCA) was employed to explore the structural and energy fluctuations on MD simulation data. Different multivariate energy factors detected the existent MD trajectory variability in the low-dimensional planetary [23, 24]. The variables considered for the structural and energy factors were bond distances, bond angles, dihedral angles, planarity, van der Waals energies, and electrostatic energies [25, 26]. The simulated data were pre-processed by centering and scaling. The last 4500 ps MD trajectories were employed to disclose the variations among the dimers and complexes. The following equation reflects the PCA model:

$$X=T_{k}P_{k}^{T}+E$$

Where, *X* matrix designates multivariate factors into the resultant of two new matrices, i.e., *T*_*k*_ and *P*_*k*_; *T*_*k*_ is the matrix of scores which indicates relation among the samples; *P*_*k*_, a matrix of loadings links the variables, *k* is the number of factors existing in the model and *E* designates the matrix of residuals. The trajectory assessment was accomplished through R [27], RStudio [28], and inner developed codes. The PCA plots were prepared using the R package ggplot2 [29].

## 3. Results

### 3.1. Dimer models of TPO WT HD, TPO MT1 HD, and TPO MT2 3D structure

**Fig 1:**
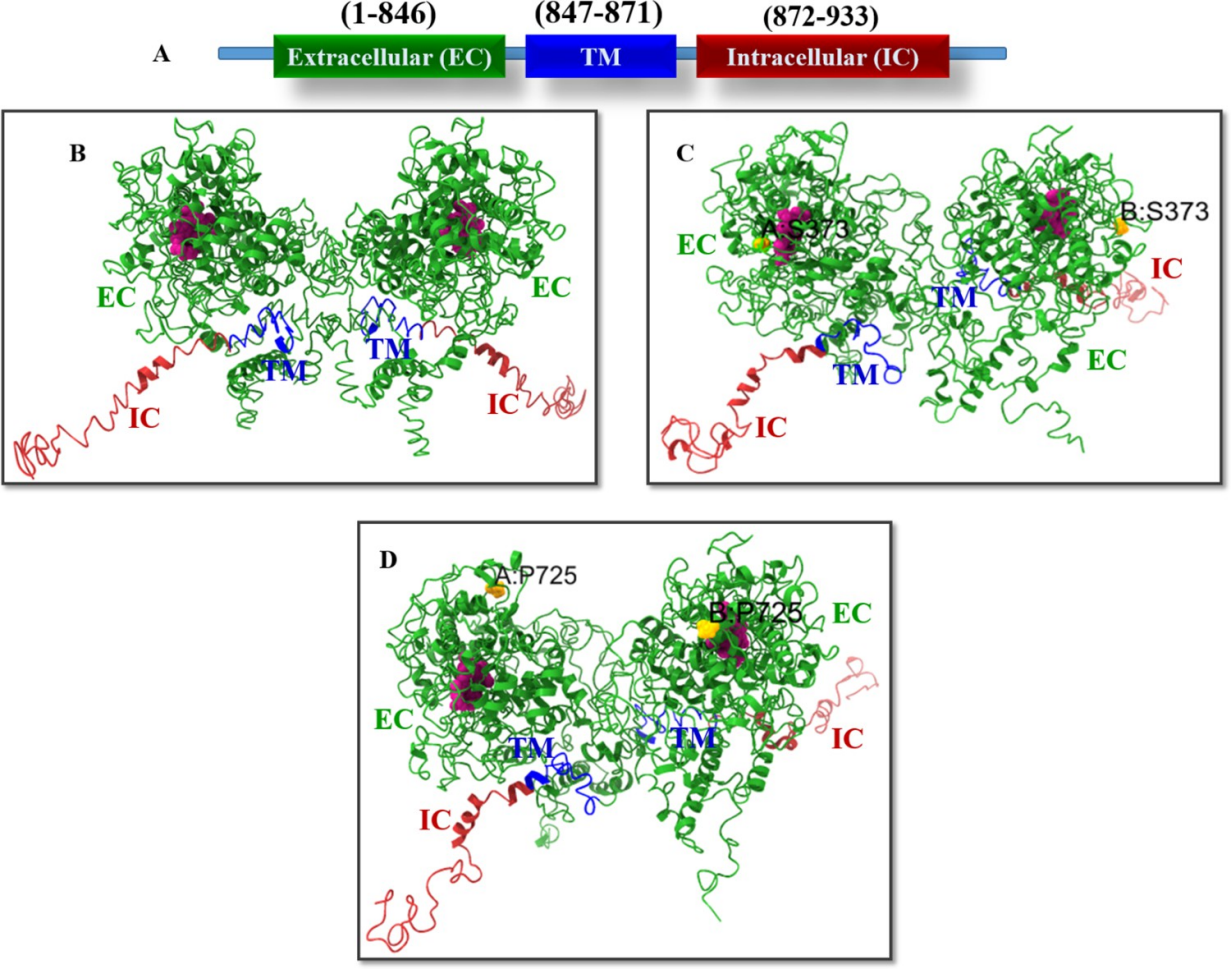
(A) TPO protein domain regions are labelled with amino acid residue numbers. (B) TPO WT HD, (C) TPO MT1 HD, and (D) TPO MT2 HD are the 3D models of the TPO homodimer proteins; heme is displayed with pink color. Mutations are marked (yellow) within each monomer of TPO MT1 HD, and TPO MT2 HD structures.

**Fig 2:**
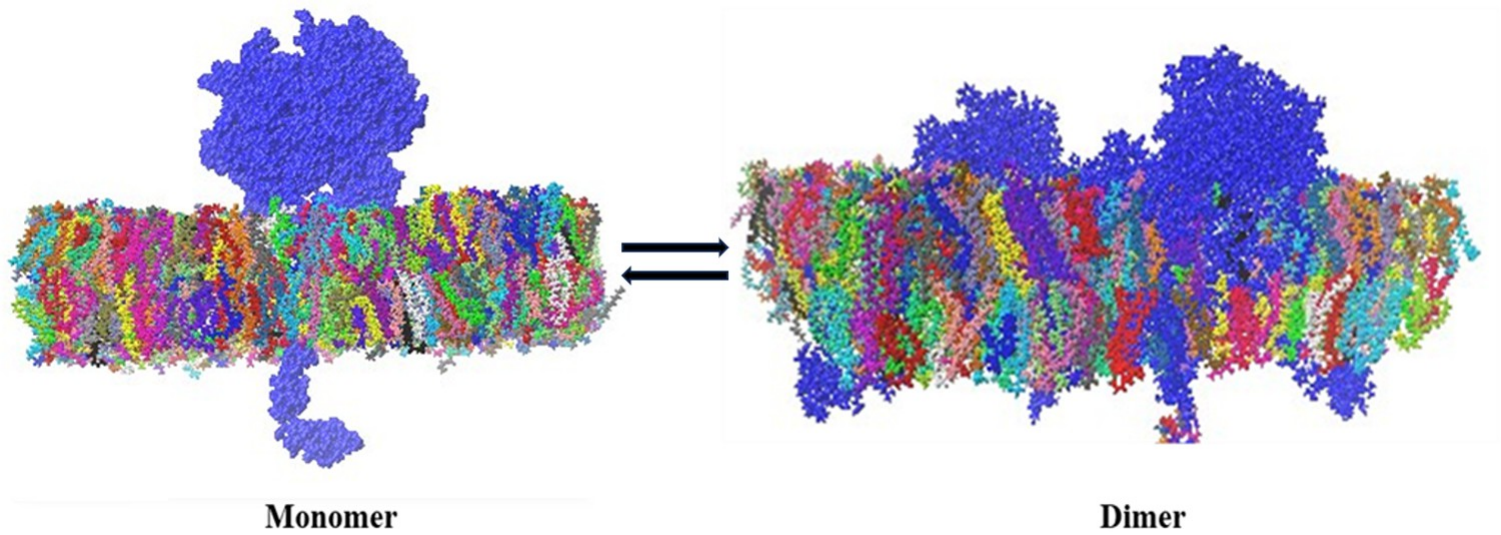
3D view of TPO protein embedded in membrane.

As TPO is a homodimer, to get dimer structure from heme docked TPO structures, we have used the SymmDock server for obtaining symmetry-based results. SymmDock server has provided TPO homodimer structures for wild type and mutants (Table 1), where docking algorithm has been applied to predict cyclically symmetric homomultimers. The Score, Area, and Atomic contact energies (ACE) values for each dimer are presented in Table 2. We have selected TPO WT dimer from the SymmDock server from those results and structural analysis, which showed the highest score is 22554 and the Area is 3691.70, and the ACE value is -361.26. TPO MT1 dimer has the highest score as 21964, and the Area is 2683.40 and ACE value is 271.65 while TPO MT2 dimer has provided highest score as 25706 and the Area is 3988.80 and ACE value is -249.80. The TPO WT HD structures, TPO MT1 HD, and TPO MT2 HD from the SymmDock server are shown in Fig 1A–1C and the dimer embedded in membrane was displayed in Fig 2.

**Table 1:** The detected mutations in *TPO* gene are considered for dimer modeling.

Exon	Position (Nucleic Acid)	Functional change (polarity)	Reference
**8**	c.1117G>T	p.Ala373Ser; (Similar to non-enzymatic reaction rate)	[2]
**12**	c.2173A>C	p.Thr725Pro; (Similar to non-enzymatic reaction rate)	[2]

**Table 2:** SymmDock server predicted structural comparison for TPO WT HD, TPO MT1 HD, and TPO MT2 HD.

Proteins	Score	Area	ACE
TPO WT HD	22554	3691.70	-361.26
TPO MT1 HD	21964	2683.40	271.65
TPO MT2 HD	25706	3988.80	-249.80

ACE = Atomic contact energies to global energies

### 3.2. Non-covalent Interactions of heme with TPO WT HD, TPO MT1 HD, and TPO MT2 HD

**Fig 3:**
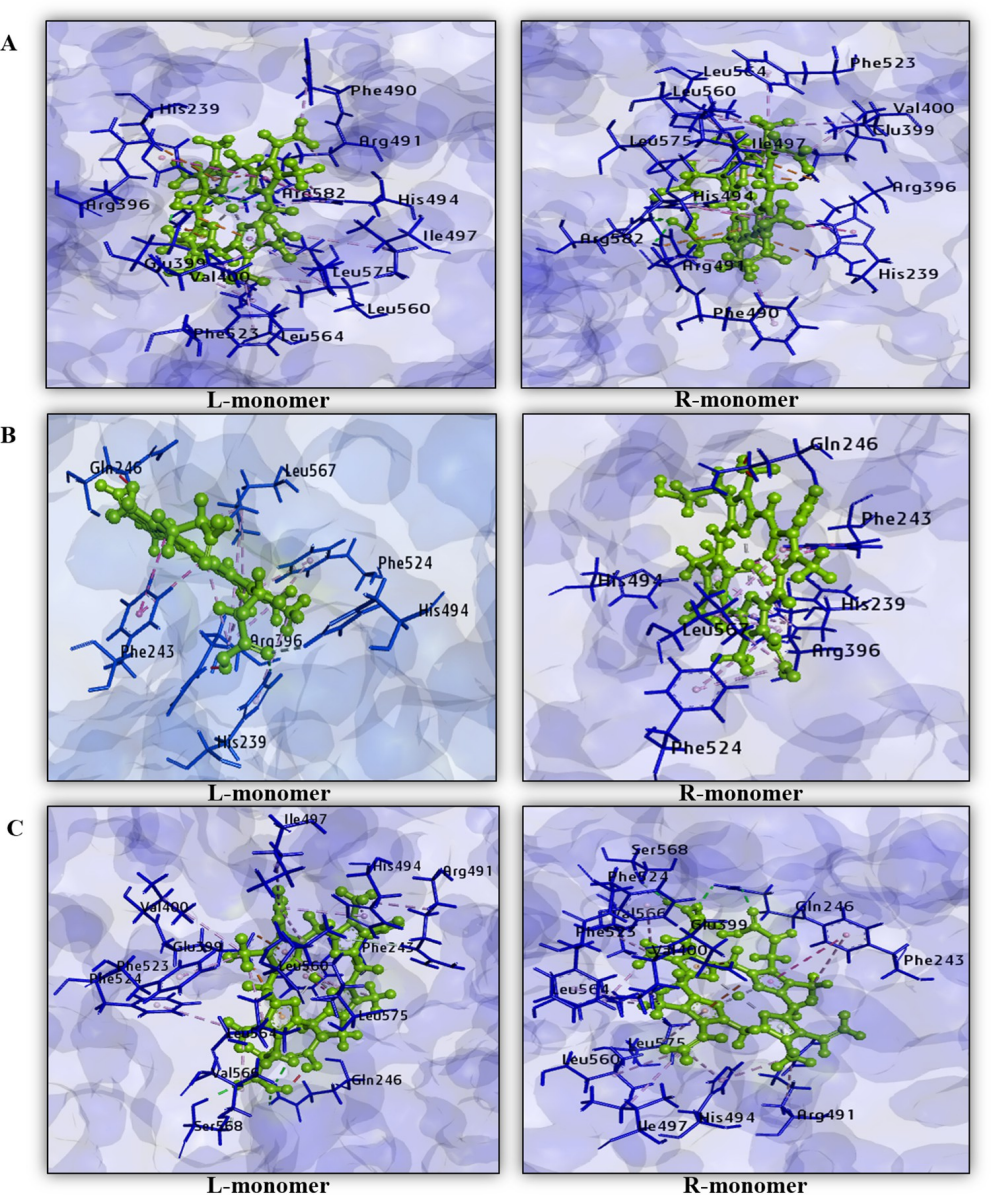
Non-covalent interactions of heme with SymmDock server predicted dimer models (A) TPO WT HD, (B) TPO MT1 HD, and (C) TPO MT2 HD among each monomer. Protein is shown by blue color and heme is shown by green color in each monomer.

The non-covalent interactions of heme with TPO WT HD, TPO MT1 HD, and TPO MT2 HD have been visualized through BIOVIA Discovery Studio version 4.5. We have analyzed the interaction of heme with wild type and mutant TPO dimer for “L (left)-monomer” and “R (right)-monomer” of each structure. The constructed comparative data tables are summarized in Table 3. Graphical presentations of non-covalent interactions are shown in Fig 3. From the data analysis, in predicted dimer structures, we have observed that heme interacts with TPO WT HD through 26 non-covalent interactions among those 4 crucial amino acids, including His239, Arg396, Glu399, and His494 are present in each monomer. Subsequently, the presence of essential residues enables TPO WT HD as enzymatically active. For TPO MT1 HD total interactions in left and right monomers are 18, respectively; among those interactions, crucial amino acids His239, Arg396 and His494 are present. Thus, in the case of TPO MT1 HD, total number of interactions is decreased significantly and also Glu399 residue, which is crucial for interaction, is absent during interactions. Hence, a similar type of damaging effect is consistent in TPO MT1 HD as in the monomer protein. In TPO MT2 HD, interaction with heme shows that each monomer participated with 25 non-covalent interactions; among those Glu399, His494 are crucial amino acids. However, the lack of His239 and Arg396, two crucial residues essential for MPO-like domain activity in the TPO enzyme, suggests that TPO MT2 HD can exert a similarly damaging effect as in the monomer. According to Begum et al., all three dimer structures TPO WT HD, TPO MT1 HD and TPO MT2 HD display similar types of crucial amino acids involved in interactions with heme comparing with TPO WT, TPO MT1, and TPO MT2 monomers, respectively [2].

**Table 3:** Non-covalent interactions of heme with SymmDock server predicted TPO WT HD, TPO MT1 HD, and TPO MT2 HD.

**TPO WT HD**				
**Monomers**	**Electrostatic bond**	**Hydrophobic bond**	**Hydrogen bond**	**Number of** **interactions**
L	Arg396, Glu399, Arg491	His239, Val400, Phe490, Arg491, His494, Ile497, Phe523, Leu564, Leu560, Leu575	Arg491, Arg582	26
R	Arg396, Glu399, Arg491	His239, Val400, Phe490, Arg491, His494, Ile497, Phe523, Leu560, Leu564, Leu575	Arg491, Arg582	26
**TPO MT1 HD**				
**Monomers**	**Electrostatic bond**	**Hydrophobic bond**	**Hydrogen bond**	**Number of interactions**
L	_	His239, Phe243, Arg396, Phe524, Leu567	His239, His494	18
R	_	His239, Phe243, Arg396, Phe524, Leu567	His239, His494	18
**TPO MT2 HD**				
**Monomers**	**Electrostatic bond**	**Hydrophobic bond**	**Hydrogen bond**	**Number of interactions**
L	Glu399	Phe243, Val400, Arg491, His494, Ile497, Phe523, Phe524, Leu560, Leu564, Val566, Leu575	Gln246, Arg491, Ser568	25
R	Glu399	Phe243, Val400, Arg491, His494, Ile497, Phe523, Phe524, Leu560, Leu564, Val566, Leu575	Gln246, Arg491, Ser568	25

### 3.3. Sequential molecular docking of TPO WT HD, TPO MT1 HD, and TPO MT2 HD with hydrogen peroxide (H_2_O_2_) and analysis of non-covalent interactions

**Fig 4:**
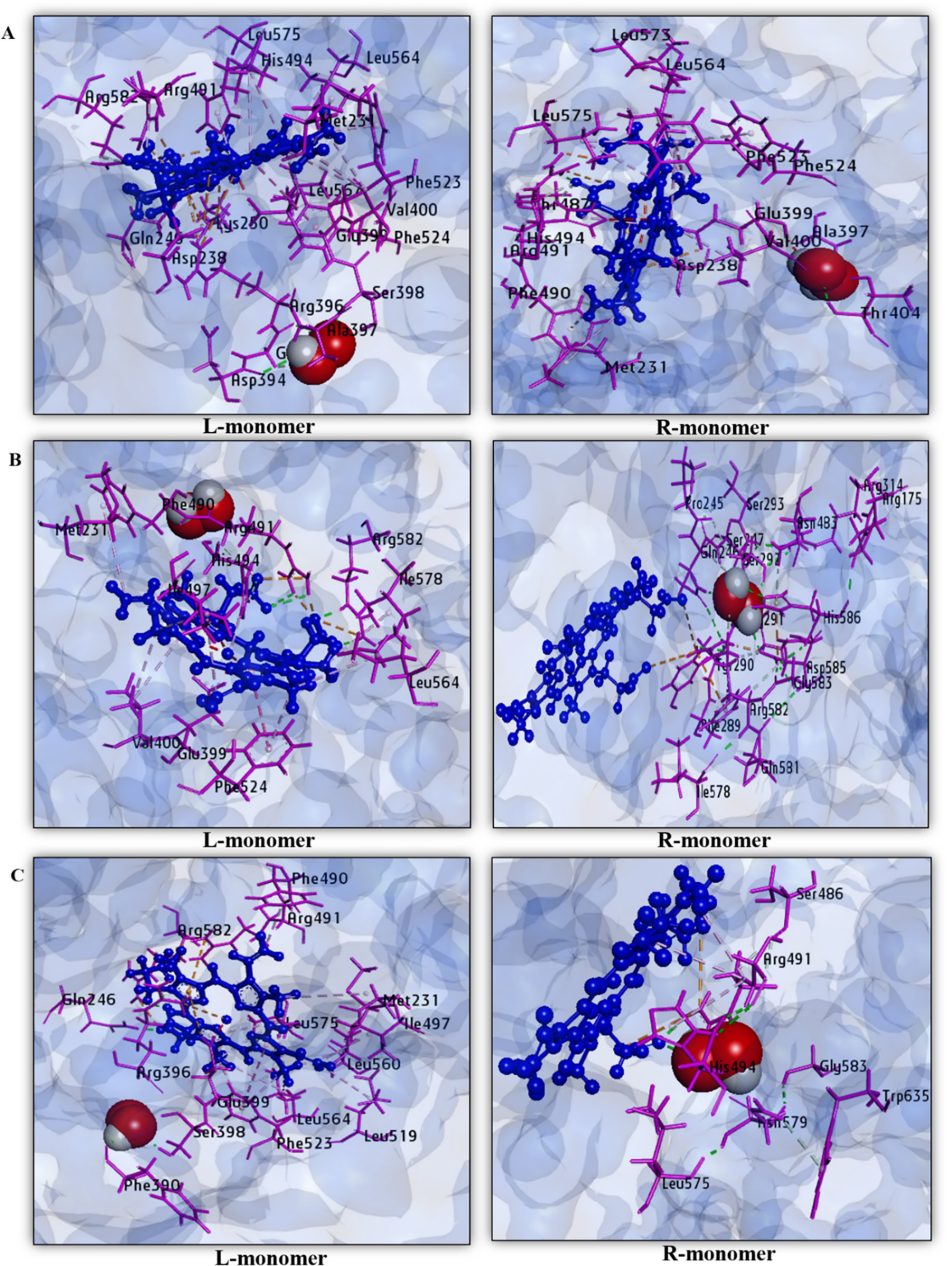
Non-covalent interactions of H_2_O_2_ with (A) TPO WT HD, (B) TPO MT1 HD, and (C) TPO MT2 HD among each monomer after sequential docking. Protein is displayed by pink color, and heme is indicated by blue color, and red-white colors display H_2_O_2_ in each monomer.

After sequential docking of H_2_O_2_ with TPO WT HD, TPO MT1 HD, and TPO MT2 HD, we have obtained the binding affinities applying AutoDock Vina protocol for “L (left)- monomer” and “R (right)-monomer” of each structure and visualized the corresponding position of H_2_O_2_ using BIOVIA Discovery Studio version 4.5. We have obtained average binding affinity for TPO WT is -2.85 kcal/mol. On the other hand, TPO MT1 HD has shown the average binding affinity as -2.65 kcal/mol. In the case of TPO MT2 HD, we have observed the the average binding affinity for H_2_O_2_ is -2.75 kcal/mol. To investigate the non-covalent interactions among H_2_O_2_ and dimer structures, we have made data tables and graphical presentations of the interacting amino acids in hydrophobic bond, hydrogen bond, and electrostatic bond (Table 4 and Fig 4). After analyzing the interaction data, we observed that TPO WT HD interacts with H_2_O_2_ through 28 interactions in the left monomer, including Gln246, Asp394, Gly395, Ser398, Arg491, Arg582 by hydrogen bond, Met231, Val400, Arg491, His494, Phe523, Phe524, Leu564, Leu567, and Leu575 by hydrophobic bond, and Asp238, Lys250, Arg396, Arg491, and Arg582 by the electrostatic bond. There are 3 crucial amino acid residues present where Asp238, Arg396 interact through electrostatic bond and His494 interacts through the hydrophobic bond. In the right monomer, there are 23 non-covalent interactions in which Gln246, Asp394, Gly395, Ala397, Ser398, Thr404, Thr487, Arg491, and Arg582 interact by hydrogen bond, Met231, Val400, Phe490, His494, Phe523, Phe524, Leu564, Leu573, and Leu575 are involved with hydrophobic bond and Asp238, Glu399, 2 crucial amino acids interact by the electrostatic bond. TPO MT1 HD interacts with H_2_O_2_ in the left monomer through 20 non-covalent interactions where Arg491, His494, and Arg582 participate by hydrogen bond, Met231, Val400, His494, Ile497, Phe524, Leu564, and Ile578 participate by hydrophobic bond and Arg491, Arg582 contribute an electrostatic bond. Only His494 is present as crucial amino acid but others Asp238, His239, Arg396, and Glu399 are absent during interactions. In the right monomer of MT1 HD interacts with 31 non-covalent interactions, among which Arg175, Pro245, Gln246, Ser247, Asn483, Tyr290, Arg291, Ser292, Ser293, Arg314, Ile578, Gln581, Arg582, Gly583, Asp585, and His586 interact through a hydrogen bond, Phe289, Arg582 interact through hydrophobic bond and Phe289, Arg582, Asp585, and His586 interact through the electrostatic bond. However, there are no crucial amino acids present in those interactions.

**Table 4:** Binding affinity and non-covalent interactions of H_2_O_2_ with TPO WT HD, TPO MT1 HD, and TPO MT2 HD protein after flexible sequential docking.

		**TPO WT HD-H**_**2**_ **O**_**2**_				
			
**Monomers**	**Binding affinity (kcal/mol)**	**Electrostatic bond**	**Hydrophobic bond**	**Hydrogen bond**	**Avg. Binding affinity (kcal/mol)**	**Number of interacts**
L	-2.8	Asp238, Lys250, Arg396, Arg491, Arg582	Met231, Val400, Arg491, His494, Phe523, Phe524, Leu564, Leu567, Leu575	Gln246, Asp394, Gly395, Ser398, Arg491, Arg582	-2.85	28
R	-2.9	Asp238, Glu399,	Met231, Val400, Phe490, His494, Phe523, Phe524, Leu564, Leu573, Leu575	Gln246, Asp394, Gly395, Ala397, Ser398, Thr404, Thr487 Arg491, Arg582		23
**TPO MT1 HD-H**_**2**_ **O**_**2**_						
**Monomers**	**Binding affinity (kcal/mol)**	**Electrostatic bond**	**Hydrophobic bond**	**Hydrogen bond**	**Avg. Binding affinity (kcal/mol)**	**Number of interacts**
L	-2.7	Arg491, Arg582	Met231, Val400, His494, Ile497, Phe524, Leu564, Ile578	Arg491, His494, Arg582	-2.65	20
R	-2.6	Phe289, Arg582, Asp585, His586	Phe289, Arg582	Arg175, Pro245, Gln246, Ser247, Tyr290, Arg291, Ser292, Ser293, Arg314, Asn483, Ile578, Gln581, Arg582, Gly583, Asp585, His586		31
**TPO MT2 HD-H**_**2**_ **O**_**2**_						
**Mono mers**	**Binding affinity (kcal/mol**	**Electrostatic bond**	**Hydrophobic bond**	**Hydrogen bond**	**Avg. Binding affinity (kcal/mol)**	**Number of interacts**
L	-2.6	Arg396, Glu399, Arg491, Arg582	Met231, Phe490, Ile497, Leu519, Phe523, Leu560, Leu564, Leu575	Gln246, Phe390, Ser398, Arg491, Arg582	-2.75	25
R	-2.9	Arg491	Arg491, His494	Ser486, Arg491, His494, Leu575, Asn579, Gly583, Trp635		16

In the case of TPO MT2 HD, the left side monomer interacts with total 25 interactions where Gln246, Phe390, Ser398, Arg491, and Arg582 display hydrogen bond, Met231, Phe490, Ile497, Leu519, Phe523, Leu560, Leu564, and Leu575 display hydrophobic bond and Arg396, Glu399, Arg491, and Arg582 exhibit electrostatic bond. The structure interacts with H_2_O_2_ through only Arg396, Glu399, 2 crucial amino acids. In the right-side monomer, this MT2 HD structure includes 16 interactions, and among those Ser486, Arg491, His494, Leu575, Asn579, Gly583, and Trp635 interact through a hydrogen bond, Arg491, His494 participate through the hydrophobic bond, and Arg491 participates through the electrostatic bond. In that structure, only His494 crucial amino acid interacts via hydrogen bond.

Consequently, TPO MT1 HD and TPO MT2 HD catalytic activity are diminished severely for the interaction with H_2_O_2._ By analyzing binding affinities and interaction between H_2_O_2_ and dimer structures, we have found that although TPO MT1 HD and TPO MT2 HD dimers show slight differences in binding affinities with TPO WT HD for H_2_O_2_, typically lack crucial amino acids to exert enzymatic activity. Among the three structures, TPO MT1 HD lacks Asp238, His239, Arg396, and Glu399, 4 crucial amino acids, and comparatively, it has showed a less binding affinity for interaction with H_2_O_2_. Thus, in comparison with monomer structures, it can be inferred that the effect of the mutation on TPO dimer is clearly damaging in the absence of interaction with catalytic site residues.

### 3.4. Sequential molecular docking of TPO WT HD, TPO MT1 HD, and TPO MT2 HD with iodide (I^−^) and analysis of non-covalent interactions

**Fig 5:**
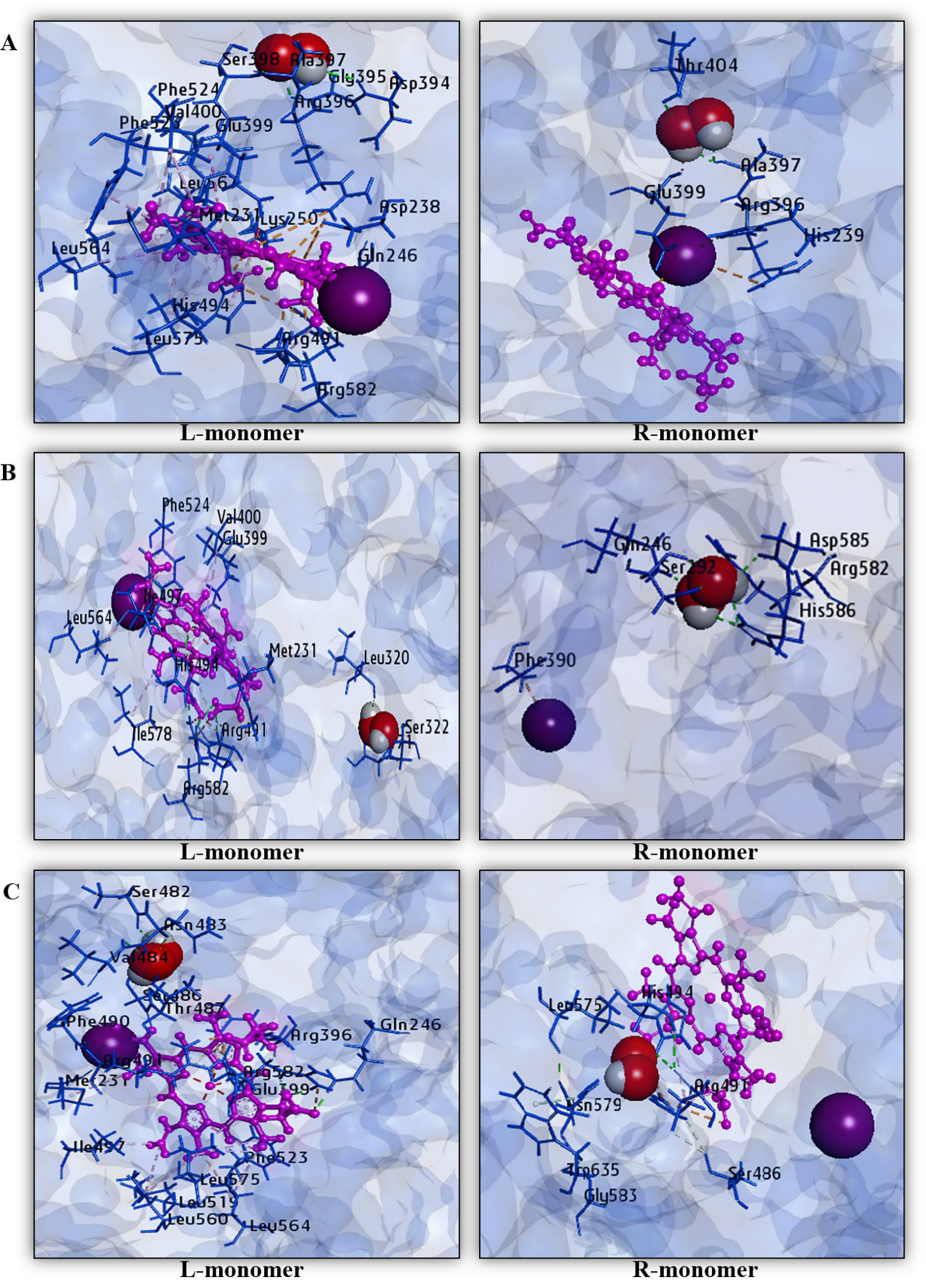
Non-covalent interactions of iodide (I^-^) with (A) TPO WT HD, (B) TPO MT1 HD, and (C) TPO MT2 HD among each monomer after sequential docking. Protein is presented by blue color and heme is presented by pink color, H_2_O_2_ is presented by red-white colors, and iodide (I^-^) is presented by violet color in each monomer.

From sequential docking of iodide (I^-^) with TPO WT HD, TPO MT1 HD, and TPO MT2 HD, we have obtained the binding affinities employing AutoDock Vina protocol for “L (left)- monomer” and “R (right)-monomer” of each structure and visualized the corresponding position of I^−^ using BIOVIA Discovery Studio version 4.5. We have obtained the average binding affinity is -1.2 kcal/mol for wild-type TPO homodimer. TPO MT1 HD shows the average binding affinity is -1.12 kcal/mol. In the case of TPO MT2 HD, we have observed the average binding affinity for MT2 homodimer also remains -1.1 kcal/mol. Consequently, there is a little change of binding affinities for I^−^ ligand in wild type and mutant TPO dimer proteins (Table 5). To determine the non-covalent interactions among I^−^ and dimer structures, we have made graphical presentations of the interacting amino acids in hydrophobic bond, hydrogen bond, and electrostatic bond. These are shown in Fig 5.

**Table 5:** Binding affinities of I^−^ with TPO WT HD, TPO MT1 HD, and TPO MT2 HD after flexible sequential docking.

Protein Monomers	TPO WT HD-I^−^ Binding Affinity (kcal/mol)	Average Binding Affinity (kcal/mol)
**L monomer**	-1.3	-1.2
**R monomer**	-1.1	
Protein Monomers	**TPO MT1 HD-I** ^**−**^ **Binding Affinity** **(kcal/mol)**	**Average** **Binding Affinity** **(kcal/mol)**
**L monomer**	-1.1	-1.12
**R monomer**	-1.13	
**Protein Monomers**	**TPO MT2 HD-I** ^**−**^ **Binding Affinity** **(kcal/mol)**	**Average** **Binding Affinity** **(kcal / mol)**
**L monomer**	-1.1	-1.1
**R monomer**	-1.1	

From the non-covalent interaction data, we have observed that most of the cases for left monomers of wild type and mutant structures show that I^−^is attached with carbon atom also displayed as carbon-bonded halogen. Besides, H_2_O_2_ has been dislocated from TPO MT1 HD left monomer, and the heme molecule is missing from the right monomer during non-covalent interaction. However, the right monomer of TPO WT HD shows interaction with I^−^ in the presence of His239 and Arg396, 2 crucial amino acids, and other Ala 397, Thr404. But in the case of TPO MT1 HD and TPO MT2 HD right monomers do not interact with I^−^ properly rather dissociation of I^−^ is observed for mutant homodimers due to the drastic absence of crucial residues in the interactions. The prime reason behind such kind of incidences might be a mutational effect which is responsible for disruption of interaction with I^−^, as the mutation in the *TPO* gene is frequently described with mild to severe repercussions resulting in partial iodine organification defect (PIOD) to total iodine organification defect (TIOD). From the analysis, it has been observed that the severe damaging effect in TPO mutant dimer proteins is consistent with monomer structures.

### 3.5. Molecular dynamics (MD) simulation and molecular docking for protein-ligand complexes

The MD simulation of each apo dimer protein and dimer-ligand complex has been conducted for 5000 ps. Then, each dimer model’s stability and dynamics are investigated during MD simulation.

Different 3D coordinate files from 0 ps to 5000 ps are selected for redocking with ligands (H_2_O_2_, I^-^) to investigate the binding affinities during MD simulation. The binding affinities for the ligands are shown in Tables 6 and 7, which indicate dynamic nature of the structures during MD simulation. Though binding affinities fluctuate during the simulation but TPO dimer mutants show lack of interactions with catalytic residues and disruption of their interaction with the essential ligands. Hence, the binding affinity for the mutant dimers do not represent the enzymatic action.

**Table 6:** Binding affinity after flexible sequential docking of H_2_O_2_ with different coordinate files generated during MD simulation for left (L) and right (R) monomers of TPO WT HD, TPO MT1 HD, and TPO MT2 HD proteins.

Structures	Binding Affinity for H_2_O_2_ (kcal/mol)												Average Binding Affinity (kcal/mol)
	0ps		1000 ps		2000 ps		3000 ps		4000 ps		5000 ps		
	L	R	L	R	L	R	L	R	L	R	L	R	
TPO WT	-2.8	-2.7	-2.93	-2.7	-2.9	-2.83	-2.83	-2.97	-2.67	-2.97	-2.8	-2.9	-2.83
TPO MT1	-2.7	-3.03	-2.8	-2.8	-2.8	-2.7	-2.77	-2.67	-2.83	-3	-2.63	-2.57	-2.77
TPO MT2	-3.1	-3.1	-2.83	-3	-3.13	-2.86	-2.97	-2.9	-2.57	-2.9	-2.6	-2.9	-2.89

**Table 7:** Binding affinity after flexible sequential docking of I^−^ with different coordinate files generated during MD simulation for left (L) and right (R) monomers of TPO WT HD, TPO MT1 HD, and TPO MT2 HD proteins.

Structures	Binding Affinity for I^−^ (kcal/mol)												Average Binding Affinity (kcal/mol)
	0ps		1000 ps		2000 ps		3000 ps		4000 ps		5000 ps		
	L	R	L	R	L	R	L	R	L	R	L	R	
TPO WT	-1.03	-1.27	-1.1	-1.2	-1	-1.1	-1.1	-1.2	-1	-1.1	-1.3	-1.1	-1.125
TPO MT1	-1.1	-1.13	-1.1	-1.2	-1.1	-1.07	-1.15	-1.1	-1	-1.2	-1.07	-1.3	-1.126
TPO MT2	-1.1	-1.1	-1	-1	-1.1	-1	-1.1	-1.1	-1	-1	-1.3	-1.1	-1.075

**Fig 6:**
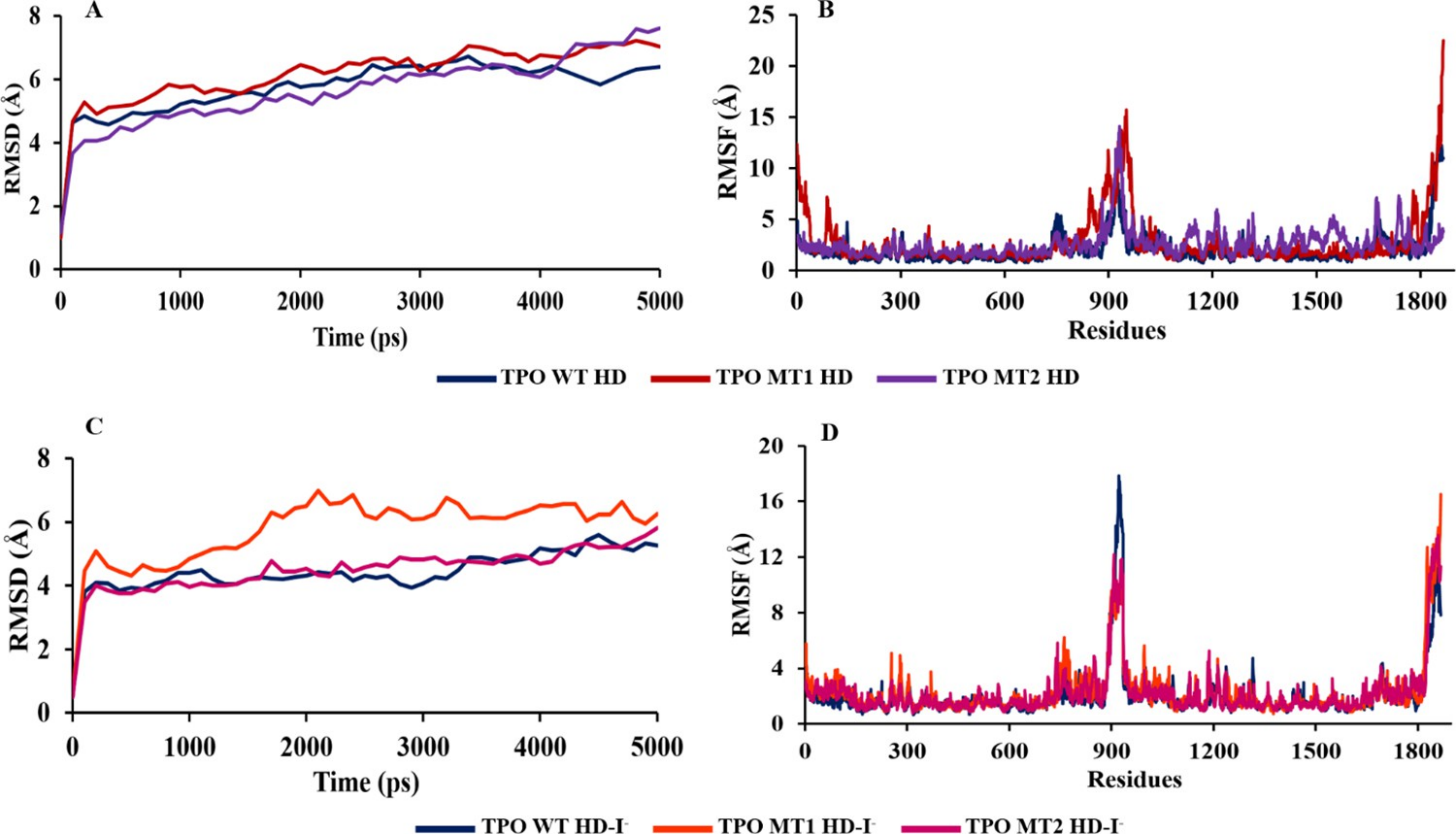
Analysis of 5000 ps MD simulation data for RMSD and RMSF values (A, B) TPO apo-dimers and (C, D) TPO dimer-ligand complexes.

The RMSD value (1.108–6.719 Å) for α-carbon atoms is lower in TPO WT HD than TPO MT1 HD and TPO MT2 HD simulation. However, TPO WT HD and TPO MT2 HD manifest a close pattern for RMSD values (⁓5.7 Å) from 3600 ps to 4000 ps. A significant deviation has been observed for TPO MT1 HD with a maximum 7.2 Å. TPO WT HD has achieved more stability than mutant TPO dimers from 4000 ps to 5000 ps (Fig 6A). RMSF value analysis also displays residues in extracellular, TM, and intracellular regions have significant variances in fluctuation among wild-type and mutant TPO dimers (Fig 6B). TPO MT1 HD exhibits the highest residual instability at positions 1–147, 847–975, 952 (15.75 Å), and 1866 (22.5 Å) compared to TPO WT HD and TPO MT2 HD. However, TPO MT2 HD shows higher deviations at 1138–1766 residues than TPO WT HD and TPO MT1 HD. Moreover, TPO WT HD manifests lower variations to the catalytic site residues Asp238, His239, Arg396, Glu399, and His494 in both monomers compared to mutants. Hence, the less RMSF fluctuation provides higher structural stability, thus, more vital catalytic interactions of wild-type dimers in physiological environments. Overall, MD simulation suggests that wild type TPO dimer is more stable than mutant dimers. This information helps further docking of H_2_O_2_ and I^−^ TPO WT HD and mutant dimers (TPO MT1 HD, TPO MT2 HD) and understand the effects of nonsynonymous mutations on the structure and function of TPO dimer protein.

For dimer-ligand complexes during the simulation, RMSD value (0.526–5.604 Å) for α-carbon atoms is lower in TPO WT HD-I^−^ than both TPO MT1 HD-I^−^ (0.517–6.976 Å) and TPO MT2 HD-I^−^ (0.493–5.823 Å) in the simulation. However, TPO WT HD-I^−^ and TPO MT2 HD-I^−^ exhibit quite a close pattern for RMSD values except during 900–1300 ps and 4400–4600 ps. The high fluctuation has been observed for TPO MT1 HD-I^−^ complex. Conversely, TPO WT HD-I^−^ (average 4.421 Å) has achieved more stability than mutant TPO dimer-ligand complexes (RMSD values average 5.783 Å and 4.763 Å) in the whole simulation (Fig 6C).

The RMSF analysis shows residual fluctuations for TPO WT HD-I^−^ intracellular region at 924 (17.47 Å) and extracellular region residues 1314–1319 (Fig 6D). However, the whole TPO WT HD-I^−^ the complex remains stable in the MD simulation system than mutant complexes. Besides, TPO wild type-ligand complex manifests lower deviations to the catalytic site residues in both monomers compared to mutant ones. Hence, the more vital catalytic interaction of wild-type dimer-ligand complex is visible in the simulation process. Overall, MD simulation suggests that the wild-type TPO dimer-ligand complex is more stable than mutants.

**Fig 7:**
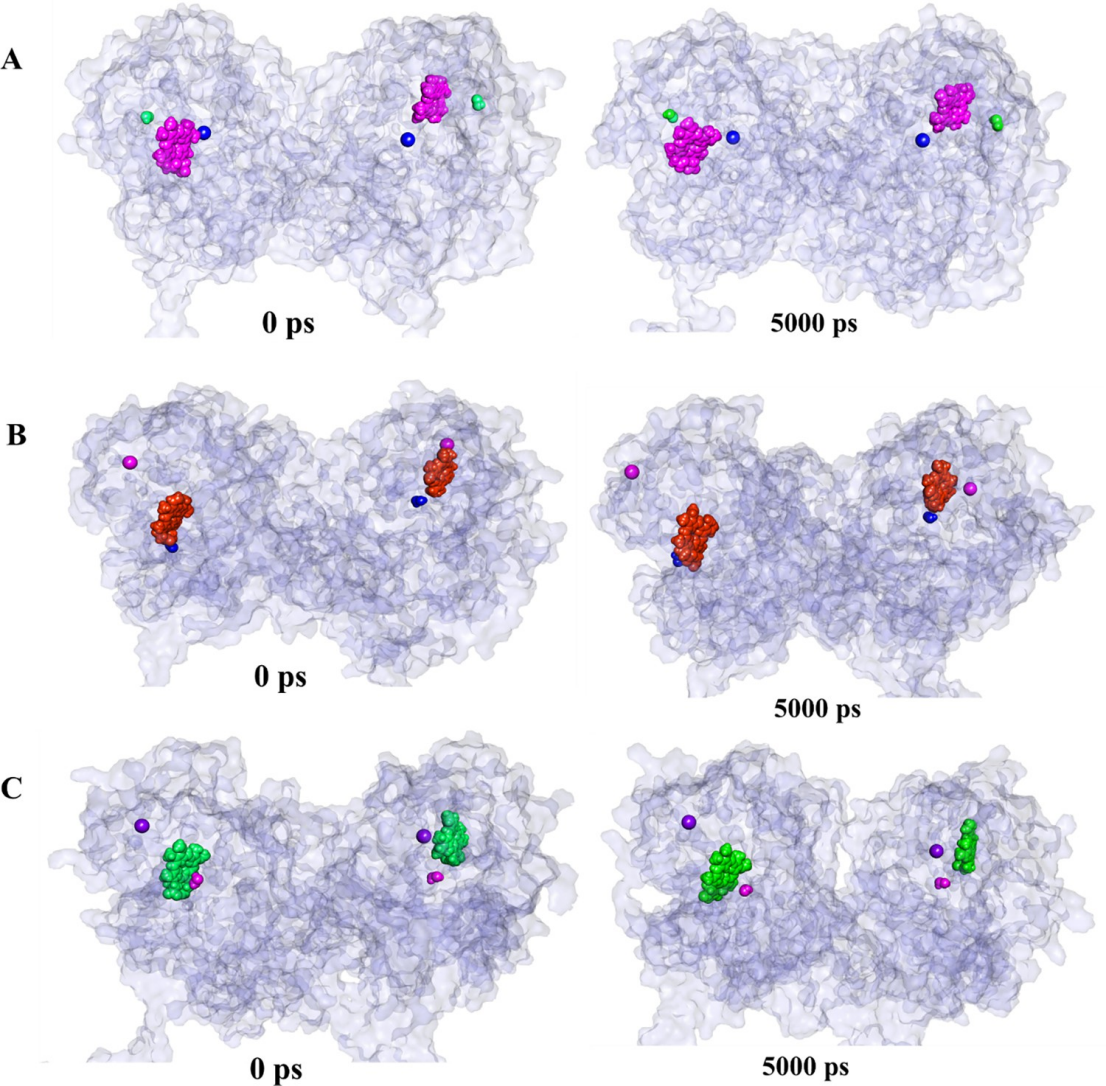
The binding poses of heme, H2O2 and I- within the TPO WT HD-I^−^, TPO MT1 HD-I^−^, and TPO MT2 HD-I− proteins over 5000 ps MD simulation. Proteins are represented by white color. (A) in TPO WT HD-I^−^, heme is displayed by pink color, H_2_O_2_ is indicated by green color, and I^-^ is shown by blue color (B) in TPO MT1 HD-I^−^, heme is displayed by orange color, H_2_O_2_ is indicated by blue color, and I^-^ is shown by pink color (C) in TPO MT2 HD-I^−^, heme is displayed by green color, H_2_O_2_ is indicated by pink color, and I^-^ is shown by violet colors.

The dynamics of snapshot conformers exhibit different binding poses of ligands among wild type and mutant dimers (Fig 7A–7C). In the case of both TPO MT1 HD-I^−^ (Fig 7B) and TPO MT2 HD-I^−^ (Fig 7C), the ligands have significantly changed their position and orientation during MD simulation. In comparison with wild-type, ligands in TPO MT1 HD-I^−^ complex display a significant shift than TPO MT2 HD-I^−^. Consequently, the effect of mutation is perceptible for mutant TPO dimers.

**Fig 8:**
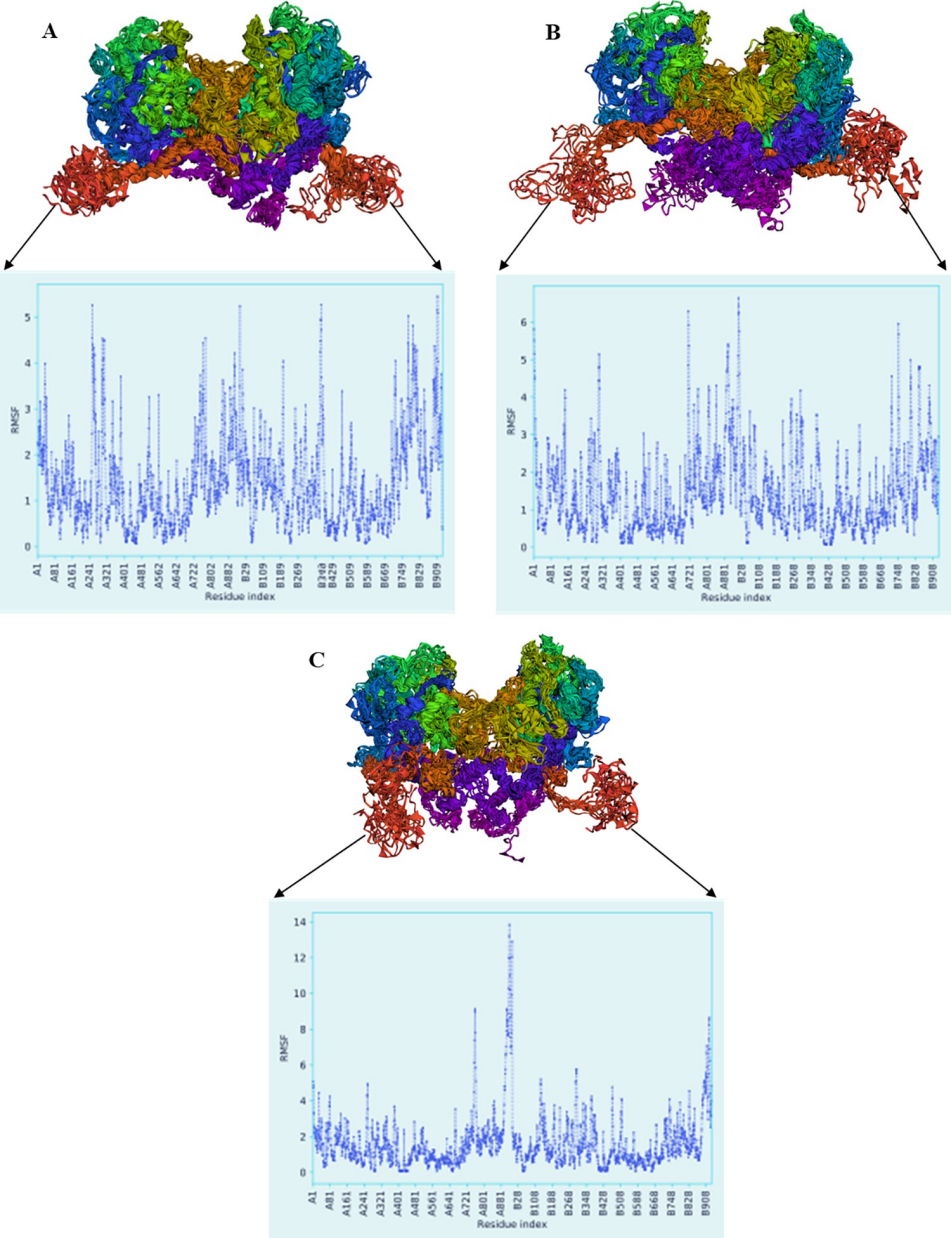
CABS-flex 2.0 simulated structures and RMSF values over 50 cycles. (A) TPO WT HD-I^-^, (B) TPO MT1 HD-I^-^, and (C) TPO MT2 HD-I^-^.

Moreover, the flexibility of protein conformers for wild type and mutant dimer-ligand complexes has been evaluated for more 50 cycles by CABS-flex 2.0 (Fig 8). The contact-maps for the residue-residue interactions of the retrieved models are shown in Fig S3 in S1 File. The superimposed structures of the 10 retrieved models for each structure are displayed in Fig 8A–8C. The RMSF plots have exposed the residual fluctuation of the structure models up to 5.5 Å for wild type, 6.6 Å for MT1, and 13.8 Å for MT2 (Fig 8A–8C). During dynamics simulation, mutant dimers are more flexible than wild type dimer which is consistent within the analyses and other studies [30–33].

### 3.6. Principal component analysis (PCA)

**Fig 9:**
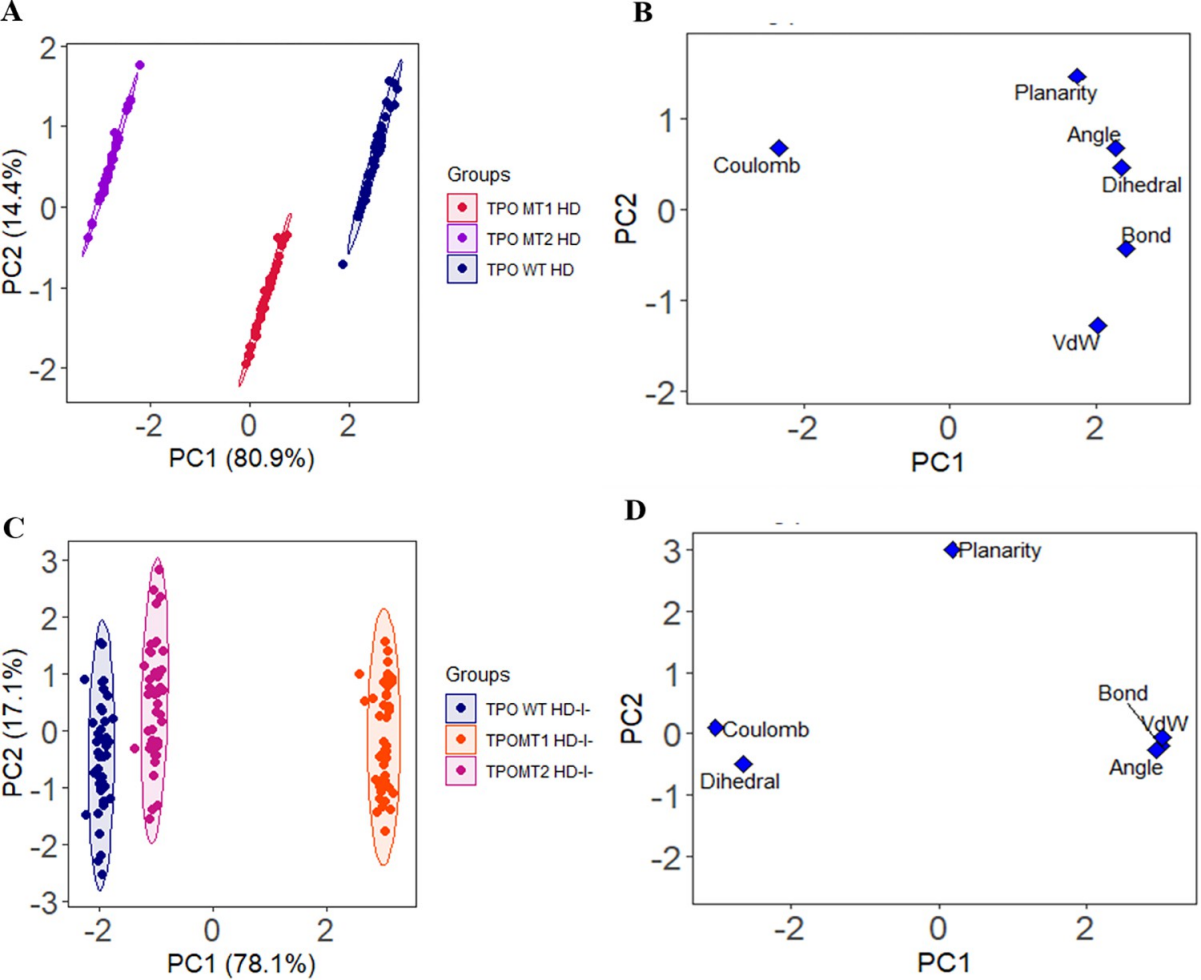
PCA of 5000 ps MD simulation data. The score plot represents three clusters for (A) TPO apo dimers and (C) dimer-ligand complexes, where each dot specifies one-time point. The clustering is attributable as TPO dimers:- TPO WT HD (blue), TPO MT1 HD (red), TPO MT2 HD (violet); dimer-ligand complexes:- TPO WT HD-I^-^ (blue), TPO MT1 HD-I^-^ (orange), and TPO MT2 HD-I^-^ (pink). (B, D) The loading plot displays the energy and structural variables from principal components analysis for TPO apo-dimers and dimer-ligand complexes accordingly.

The energy and structural data of simulated apo-dimer proteins (TPO WT HD, TPO MT1 HD, and TPO MT2 HD) and the dimer-ligand complexes (TPO WT HD-I^-^, TPO MT1 HD-I^-^, and TPO MT2 HD-I^-^) are evaluated by principal component analysis (PCA). Here, we have visualized the actual differences among wild-type and mutant proteins during MD simulation. The scores plots have disclosed the distance among the wild type and mutant dimer proteins and dimer-ligand complexes. In apo-dimers, TPO WT HD and TPO MT2 HD are remotely situated, while TPO MT1 HD resides beside the wild-type protein (Fig 9A). These visible variations indicate that the bond, bond angle, dihedral angle, planarity, coulombic energies are lowest in TPO MT2 HD however, van der Waals energies are highest. The loading plot exhibits the relationship among the variables for wild type and mutant dimers, where coulomb is much different than others (Fig 9B). Overall, 95.3% of the variance has been disclosed by PC1 and PC2, where PC1 expresses 80.9%, and PC2 depicts 14.4% of the variance for apo-dimers.

Conversely, a total 95.2% variance has been exposed by PC1 and PC2, where PC1 expresses 78.1%, and PC2 discloses 17.1% of the variance for dimer-ligand complexes. TPO WT HD-I^-^ and TPO MT1 HD-I^-^ are distantly located in their complex forms (Fig 9C); higher deviations during MD simulation as well lowest catalytic activity are relevant for the differences. However, TPO MT2 HD-I^-^ is located nearby to TPO WT HD-I^-^ which indicating a close structural and energy profile between both complexes. The loading plot (Fig 9D) demonstrates that bond, bond angles, van der Waals energies are closely distributed hence displays a more substantial relationship among the variables. Moreover, these variables show an increased pattern in TPO MT1 HD-I^-^ compared to TPO WT HD-I^-^ and TPO MT2 HD-I^-^. After evaluating PCA models, mostly mutant dimers show much more variations in apo and complex forms than wild type due to higher discrepancies of mutant dimers in their structural and energy trajectories during the simulation.

## 4. Discussion

The insight into the effect of nonsynonymous mutations on TPO dimer’s structural features and its biological function is crucial to reveal the severity of congenital hypothyroidism. However, genomic variation raises daunting biomedical research challenges to describe how the disease develops at the molecular and cellular levels [34].

From our previous analysis, two nonsynonymous mutations, including p.Ala373Ser and p.Thr725Pro in exons 8 and 12 of the *TPO* gene have been detected to be pathogenic or disease-causing mutations among the 36 TDH patients [2]. By considering those aspects, after a sequence-based study, the effect of the detected mutations has been investigated for mature TPO dimer through *in silico* approach among congenital hypothyroid patients in Bangladesh. The present study performed modeling of the three-dimensional (3D) structures of TPO (Wild type and Mutants) homodimer protein applying the symmetry-based algorithm of the Symmdock server. We targeted full-length homodimers due to a better understanding of the outcome of TPO full-length monomers [2]. Here, the mutational effects on TPO homodimer are firstly observed through non-covalent interactions of heme in each monomer of wild type and mutant dimers. After the analysis, mutant dimers displayed a lack of interactions with catalytic residues, and a similarly damaging effect is persistent in the mutant dimer structures like mutant monomers [2]. The recurrent evidence has been detected through evaluating RMSD and RMSF data where mutant TPO MT1 HD and TPO MT2 HD structures exert less structural stability than TPO WT HD. The higher fluctuations in crucial amino acids of mutant dimers imply less interactions with heme as well as a drastic change in enzymatic activity compared to wild-type TPO dimer.

Then we used the MD simulated dimer structure for further analyses. The enzymatic reaction for the formation of triiodothyronine (T3) and thyroxine (T4) hormone requires H_2_O_2_, iodide, and thyroglobulin (Tg). Further, we have explored H_2_O_2_ and iodide (I^-^) interactions simultaneously with the wild-type and mutant dimer to investigate the mutations’ effect on the thyroid hormone synthesis pathway’s enzymatic process. From the sequential docking analysis, we observed that TPO WT homodimer (HD) showed a comparatively higher binding affinity for H_2_O_2_ than mutant dimers; among the structures TPO MT1 HD showed the lowest binding affinity. From the analysis of non-covalent interactions, it is clear that TPO MT1 HD and TPO MT2 HD’s catalytic activity is reduced severely for the interaction with H_2_O_2._ Due to lack of interaction with crucial amino acids, TPO MT1 HD and TPO MT2 HD dimers typically fail to exert enzymatic activity. Among those three structures, TPO MT1 HD lacks interactions for Asp238, His239, Arg396, and Glu399, 4 crucial amino acids. Still, in TPO MT2 HD, 3 interactions with Arg396, Glu399, and His494, and in TPO WT HD, 4 interactions with Asp238, His239, Arg396, and Glu399 are visualized for dimer-H_2_O_2_ complexes. Nevertheless, in the case of iodide (I^-^), the sequential docking result is also more interesting. Iodide (I-) binding affinity in the three structures are close to each other, but the TPO WT HD displayed slightly higher while TPO MT1 HD and TPO MT2 HD showed a little less binding affinity for interaction. Most of the cases in left monomers of wild type and mutant dimers show that iodide (I^-^) is attached with a carbon atom and form carbon-bonded halogen. Meanwhile, halogens display anisotropic electron charge distribution but, not all docking programs constitute halogen bonding scoring functions [35]. Moreover, H_2_O_2_ is displaced from TPO MT1 HD left monomer, and the heme molecule is absent in non-covalent interaction for the right monomer. Conversely, the right monomer of TPO WT HD displays interaction with I^-^ in the presence of His239 and Arg396, 2 crucial amino acids, and other Ala 397, Thr404. While due to severe deficiency of essential residues, TPO MT1 HD and TPO MT2 HD right monomers do not interact with I^−^ accurately rather detachment of I^−^ has been witnessed in mutant homodimers. The incidences imply mutational effect, as TPO dimer shows reduced enzymatic acitivity in mutants for interaction with heme which is responsible for subsequent disruption of interaction with I^-^. Hence, the function of TPO dimer protein is affected by mutations thereby interrupt their interactions with ligands (heme, H_2_O_2_, I^-^) and regulated by other cellular processes, e.g., post-translational processes, intracellular trafficking, and targeting. After the MD simulation, it is observed that TPO WT HD-I^−^ (RMSD value: average 4.421 Å) has displayed more stability than mutant (MT1 and MT2) TPO dimer-ligand complexes (RMSD values: average 5.783 Å and average 4.763 Å) in the whole simulation. In RMSF data, the TPO wild-type dimer-ligand complex manifests lower deviations to the catalytic site residues, thus exerting stronger catalytic interactions with ligands than mutant ones. As a whole, MD simulation proposes that the wild-type TPO dimer-ligand complex is more stable than mutants. Moreover, the snapshots illustrate the corresponding position and orientation of heme, H_2_O_2,_ and I^-^ are significantly changed in both TPO MT1 HD-I^−^ and TPO MT2 HD-I^−^ after MD simulation compared to TPO wild type dimer. Later, CABS-flex 2.0 web server also revealved the flexibility of protein conformers. The mutant dimers are more flexible and unstable than wild type dimer which is supported by other studies [2, 30–32].

These differences have been emphasized by PCA model assessment, in which typically mutant dimers as apo and complex forms both show much discrepancies than wild type due to their higher transformations in their structural and energy profiles during the simulation. Subsequently, the disease phenotype may arise, disrupting the enzymatic activity to interact with crucial partner molecules [36]. The genomic defect in *TPO* leads to the following circumstances: TPO cannot bind heme molecule, TPO is unable to bind with iodide (I^−^) or thyroglobulin, and abnormal TPO direct to wrong cell membrane localization [37], which are supportive to our findings. Another important mechanism is the “dominant-negative effect,” which can cause up to 75% loss of function in a homodimer [36]. Therefore, the severe detrimental effect of mutation is distinguishable in our mutant (A373S and T725P) TPO dimers. The investigation of homodimer interactions at the atomic level is critical to realize their biological mechanisms; also, mutations result in poor thermostability [9]. Besides, *in silico*, the prediction of membrane proteins, especially dimers or high-order oligomers, remains the preliminary step [38, 39]. Consequently, despite well-known limitations, detailed inspection of a ligand in a complex with its target protein is mostly considered a treasured piece of information to understand biological phenomena. Overall, this study might be helpful for extensive knowledge on both TPO wild type and mutant dimers. As far as we know, this is the first study in our country. A comprehensive explanation has been illustrated disrupting interaction with H_2_O_2_ and iodide (I^-^) due to the effect of the nonsynonymous mutation on TPO dimer formation and its biological function for thyroid dyshormonogenesis.

## 5. Conclusion

The study was performed to investigate the effects of nonsynonymous mutation in TPO dimer formation and how it could affect its biological function in the thyroid hormone synthesis pathway. The phenomena were explored through *in silico* approach by using bioinformatics tools. As computational methods have become indispensable for studying protein-ligand, protein-protein interactions, and the structural characterization so, based on the earlier investigation of TPO monomer, we have employed sequential molecular docking, non-covalent interaction, molecular dynamics simulation, and principle component analysis to find out the structural changes due to mutations in the TPO dimer protein. From the present study, we have found that a severe damaging effect was consistent due to the mutations. Moreover, the detachment of I^−^ ligand during interaction with mutant dimers possibly results in partial iodine organification defect (PIOD) to total iodine organification defect (TIOD). Due to “dominant-negative effect” in a homodimer, loss of enzymatic activity is higher than mutant monomers. This study will be helpful in exploring the influence of other factors due to mutations involved in thyroid dyshormonogenesis and will pave the path towards the development of more efficient treatment strategies.

## Supporting information

S1 File (DOCX)
