# Supplementary material for: Investigation of the impact of nonsynonymous mutations on thyroid peroxidase dimer
Source: PLoS One. 2023 Sep 12;18(9):e0291386. doi: 10.1371/journal.pone.0291386 (PMC10497151; doi:10.1371/journal.pone.0291386)
Supplement: S1 File — (DOCX) [file pone.0291386.s001.docx]

**Investigation of the Impact of Nonsynonymous Mutations on Thyroid Peroxidase** **Dimer**

Mst. Noorjahan Begum^1,2,3^, Rumana Mahtarin^2,4^, Sinthyia Ahmed^5^, Imrul Shahriar^5^, Shekh Rezwan Hossain^2^, Md. Waseque Mia^4^, Syed Saleheen Qadri^2†^, Firdausi Qadri^2,6^, Kaiissar Mannoor^2^, Sharif Akhteruzzaman^1^*

^1^Department of Genetic Engineering & Biotechnology, University of Dhaka, Dhaka-1000, Bangladesh.

^2^Institute for Developing Science and Health Initiatives (ideSHi), ECB Chattar, Mirpur, Dhaka, Bangladesh.

^3^Virology Laboratory, Infectious Diseases Division, International Centre for Diarrhoeal Disease Research, Bangladesh, Mohakhali, Dhaka, Bangladesh.

^4^Department of Biochemistry and Molecular Biology, Shahjalal University of Science and Technology, Sylhet-3114, Bangladesh.

^5^Division of Computer Aided Drug Design, The Red-Green Research Centre, BICCB, 16 Tejkunipara, Tejgaon, Dhaka, 1215, Bangladesh.

^6^Mucosal Immunology and Vaccinology, Infectious Diseases Division, International Centre for Diarrhoeal Disease Research, Bangladesh, Mohakhali, Dhaka, Bangladesh.

^†^Deceased

*Correspondence: [sazaman@du.ac.bd](mailto:sazaman@du.ac.bd)


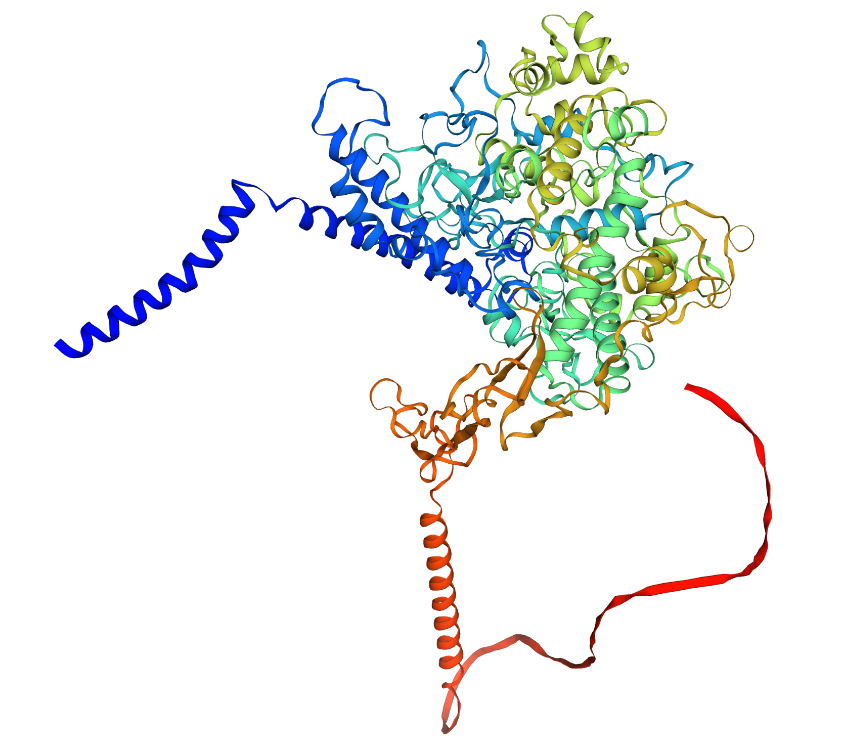


**Fig S1.** TPO monomer structure of AlphaFold and Swiss-Model web server.

**
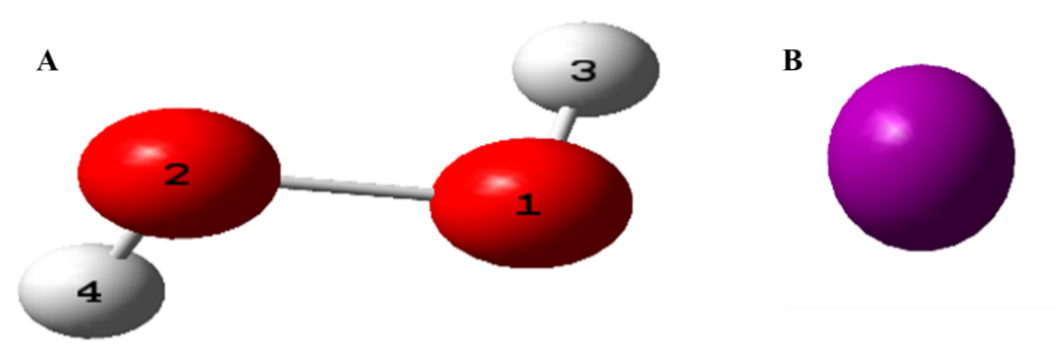
**

**Fig S2.** **Structures of ligands after optimization from different points of view.**

(A) H_2_O_2_, and (B) $I^{-}$. Figures were taken using DFT-B3LYP method and 6-31G and MIDIX basis set for H_2_O_2,_ and $I^{-}$ respectively.


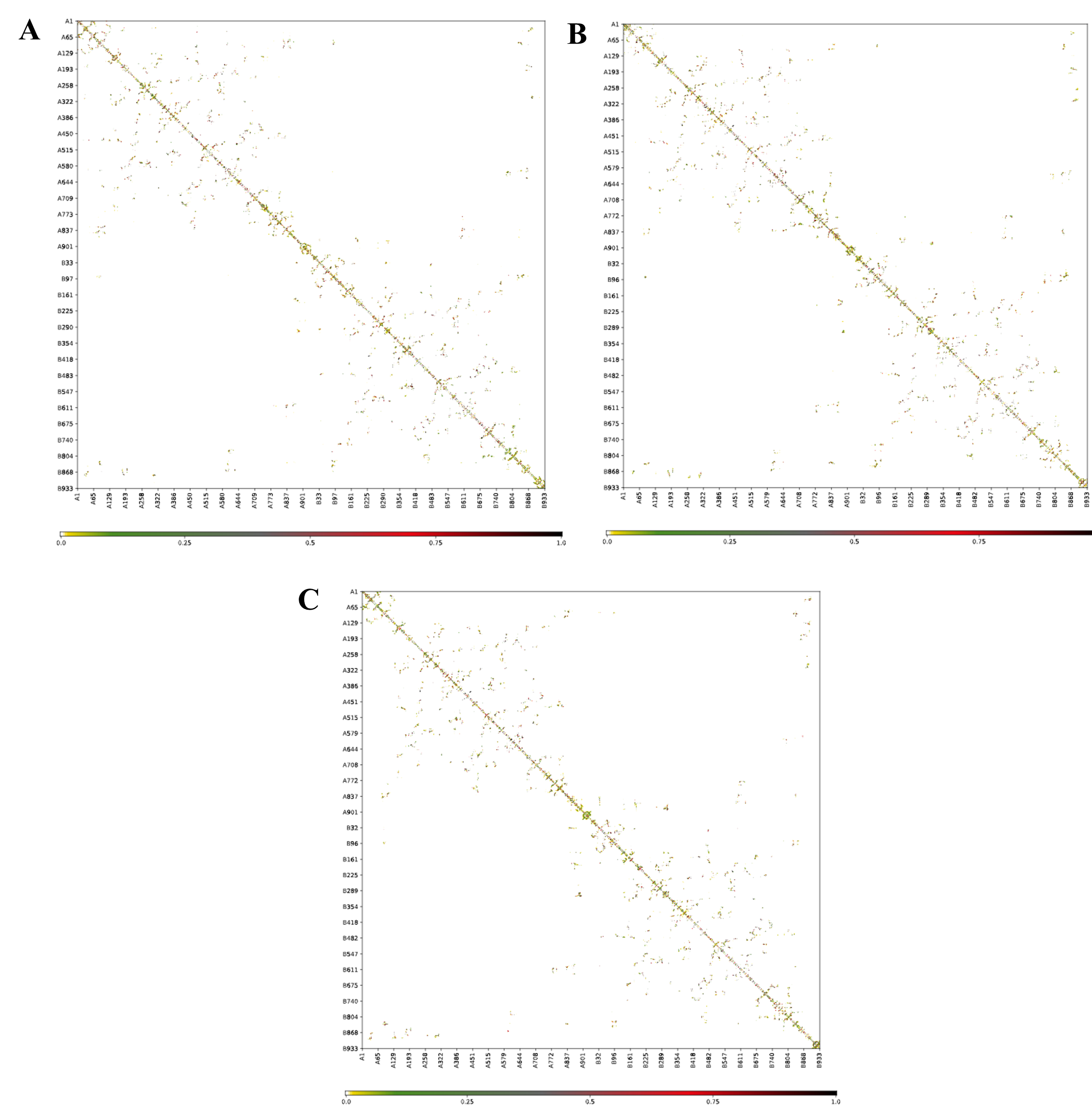


**Fig S3.** CABS-flex 2.0 simulated contact maps of the structures over 50 cycles.

1. TPO WT HD-I^-^, (B) TPO MT1 HD-I^-^, and (C) TPO MT2 HD-I^-^.

**Table S1. Summary of the Verify3D and RAMPAGE webserver results for the predicted 3D structures of TPO WT, TPO MT1, TPO MT2 proteins.**

| **Server Name** | **Region** | **TPO WT** | **TPO MT1** | **TPO MT2** | **Reference** |
| --- | --- | --- | --- | --- | --- |
| Verify3D | **_** | 73.10% | 70.74% | 76.63% | [2] |
| RAMP-AGE | Favored region | 68.40% | 69.60% | 69.80% |  |
|  | Allowed region | 17.20% | 16.20% | 18.80% |  |
|  | Outlier region | 14.40% | 14.20% | 11.40% |  |

**Table S2. The summary data for the bond distances and bond angles of specific atoms of H_2_O_2_ after optimization.**

| **Atoms** | **DFT Structure** | | |
| --- | --- | --- | --- |
|  | **Bond distance (nm)** | **Atoms** | **Bond angle (degrees)** |
| O1…H3 | 0.97 | O2…O1…H3 | 100.33° |
| O1…O2  O2…H4 | 1.48  0.97 | O2…O1…H4 | 100.33° |

**Table S3. Grid box center and the grid box size for H_2_O_2_ docking with L-monomer of TPO WT HD, TPO MT1 HD, and TPO MT2 HD**

| **Proteins** | **Grid box center (Å)** | | | **Grid box size (Å)** | | |
| --- | --- | --- | --- | --- | --- | --- |
|  | **X** | **Y** | **Z** | **X** | **Y** | **Z** |
| TPO WT | 122.7812 | 77.0660 | 106.1881 | 25.0 | 25.0 | 25.0 |
| TPO MT1 | 124.7638 | 77.3774 | 106.9257 | 25.0 | 25.0 | 25.0 |
| TPO MT2 | 127.1503 | 73.5396 | 108.7421 | 25.0 | 25.0 | 25.0 |

**Table S4. Grid box center and the grid box size for H_2_O_2_ docking with R-monomer of TPO WT HD, TPO MT1 HD, and TPO MT2 HD**

| **Proteins** | **Grid box center (Å)** | | | **Grid box size (Å)** | | |
| --- | --- | --- | --- | --- | --- | --- |
|  | **X** | **Y** | **Z** | **X** | **Y** | **Z** |
| TPO WT | 96.3495 | 63.3840 | 39.3653 | 25.0 | 25.0 | 25.0 |
| TPO MT1 | 95.0387 | 61.8934 | 42.6316 | 25.0 | 25.0 | 25.0 |
| TPO MT2 | 95.7214 | 60.9646 | 35.9866 | 25.0 | 25.0 | 25.0 |

**Table S5. Grid box center and the grid box size for** $\mathbf{I}^{\mathbf{-}}$ **docking with L- monomer of TPO WT HD, TPO MT1 HD, and TPO MT2 HD**

| **Proteins** | **Grid box center (Å)** | | | **Grid box size (Å)** | | |
| --- | --- | --- | --- | --- | --- | --- |
|  | **X** | **Y** | **Z** | **X** | **Y** | **Z** |
| TPO WT | 122.3817 | 77.7838 | 105.9090 | 24.4627 | 23.7084 | 21.8167 |
| TPO MT1 | 125.7717 | 77.9564 | 107.0975 | 22.3290 | 20.7743 | 19.0315 |
| TPO MT2 | 128.4646 | 75.0408 | 107.3137 | 25.0 | 25.0 | 25.0 |

**Table S6. Grid box center and the grid box size for** $\mathbf{I}^{\mathbf{-}}$ **docking with R- monomer of TPO WT HD, TPO MT1 HD, and TPO MT2 HD**

| **Proteins** | **Grid box center (Å)** | | | **Grid box size (Å)** | | |
| --- | --- | --- | --- | --- | --- | --- |
|  | **X** | **Y** | **Z** | **X** | **Y** | **Z** |
| TPO WT | 97.4289 | 64.1942 | 40.0406 | 25.0 | 21.7452 | 21.2454 |
| TPO MT1 | 95.7978 | 61.6505 | 41.3887 | 25.0 | 25.0 | 25.0 |
| TPO MT2 | 96.3261 | 61.2894 | 45.0320 | 24.9598 | 25.0 | 25.0 |
